# Changes Over Time and Predictors of Online Gambling in Three Norwegian Population Studies 2013–2019

**DOI:** 10.3389/fpsyt.2021.597615

**Published:** 2021-04-15

**Authors:** Ståle Pallesen, Rune Aune Mentzoni, Arne Magnus Morken, Jonny Engebø, Puneet Kaur, Eilin Kristine Erevik

**Affiliations:** ^1^Department of Psychosocial Science, University of Bergen, Bergen, Norway; ^2^Norwegian Competence Center for Gambling and Gaming Research, University of Bergen, Bergen, Norway; ^3^Optentia, The Vaal Triangle Campus of the North-West University, Vanderbijlpark, South Africa; ^4^Norwegian Gaming Authority, Førde, Norway

**Keywords:** online gambling, trend data, representative sample, predictors, mode of internet access, changes

## Abstract

**Objectives:** To investigate changes over time and identify predictors of online gambling among gamblers by using three Norwegian representative samples covering a 6-year (2013–2019) period. We also aimed to identify different characteristics (including video game participation and video gaming problems) of online compared to offline gamblers.

**Methods:** Data from gamblers (*N* = 15,096) participating in three cross-sectional surveys (2013, 2015, and 2019) based on random sampling from the Norwegian Population Registry were analyzed. Participants were asked how frequently they engaged in online gambling on different platforms (e.g., mobile phone). Data on sociodemographics, games gambled, gambling problems, gaming, and problem gaming were collected and analyzed by logistic regression analyses.

**Results:** Overall, an increase in online gambling from 2013 to 2015 was found (a larger percentage of gamblers reported having gambled online at least once during the last year), and an increase in online gambling from 2015 to 2019 was found (more gamblers reported having gambled online at least once last year and at least once per week). The increase was largest for gambling on mobile phone. Consistent predictors of online gambling (at least once last year and at least once per week) were male gender, high income, being unemployed, being on disability pension, having work assessment allowance, being a homemaker or retiree, number of games gambled, and gambling problems.

**Conclusions:** Online gambling, especially on mobile phones, has increased significantly during the last 6 years in Norway. Hence, gambling availability seems to have grown, which may pose a risk for development of gambling problems. Compared to offline gamblers, online gamblers were more likely to be men, young, not working or studying, gambling on several games, and having gambling problems. Responsible gambling efforts aiming at preventing or minimizing harm related to online gambling should thus target these groups.

## Introduction

During recent decades, we have witnessed a sharp rise in Internet use. The partial substitution of many offline activities, including gambling, with online analogs is probably both a cause and a consequence of this increase. Online gambling is however assumed to be more addictive than offline gambling, as the former entails greater availability (both in terms of time and location), anonymity, ease of betting, and enabling of games with high gambling speed (1–4). Online gambling is also cheaper to operate, often leading to higher payout ratios, which may also intensify gambling behavior. In line with this, a German study estimated that replacing 10% of offline gambling with online gambling would increase an individual's likelihood of being a problematic gambler by 8.8–12.6% (5).

So far, few studies (6, 7) have investigated which mode of access online gamblers use. However, one study of treatment-seeking gamblers showed that mobile phones were the most commonly used platform for gambling online (7). Whether the prevalence rates of online gambling through mobile phones have changed over time in line with the development of smart phone technology has previously not been investigated, hence this should be elucidated empirically.

The vast majority of studies to date show that online gamblers report more gambling problems than offline gamblers (8–14). However, one study found an inverse relationship between online gambling and gambling problems when controlling for the number of gambling activities (15). Consequently, it is recommended that controlling for the latter is important when investigating whether online gambling actually is associated with gambling problems (16–18). Another consistent finding, in addition to a higher prevalence of gambling problems among online compared to offline gamblers, is that online gambling is associated with the male gender and young subjects (9, 13, 19). The following factors have also been associated with online (as opposed to offline) gambling in at least one study: being single, consuming more alcohol, well-educated and in managerial/professional occupations, tobacco use, fewer gambling fallacies, being employed, more positive attitudes toward gambling, higher gambling expenditure, not being Asian, illicit drug use, higher household income, being engaged in a higher number of gambling activities, and being more likely to bet on sports (9, 19, 20). Still, the number of studies identifying predictors of online gambling is rather limited, and few such studies have been conducted using national representative samples of gamblers. Hence, more studies identifying characteristics of online gamblers are warranted.

Another pertinent topic in terms of online gambling concerns the relationship with video game playing. Although one study showed that consumers perceive clear market boundaries between online gambling and gaming products (21), it has nevertheless been suggested that video games with perceived gambling elements may initiate the process of normalizing and increasing the interest in gambling (22). Studies have attested to this notion, showing a positive relationship between online gambling and Internet gaming disorder (23), and a longitudinal study showed problematic video gaming to be a predictor of later problematic gambling (24). A link between problematic gambling and purchase of loot boxes in video games has also been documented (25).

Against this backdrop, the aim of the present study was to investigate changes over time and identify predictors of online gambling among gamblers by using three Norwegian representative samples covering a 6-year (2013–2019) period. We also aimed to identify different characteristics (including video game participation and video gaming problems) of online compared to offline gamblers. The following research questions were formulated: (1) Is there an overall difference in the proportion of Internet gamblers (gambling online either at least once last year and at least weekly) between 2013, 2015, and 2019? (2) Is there a difference in the proportion of gamblers using stationary computers, laptops, tablets, and mobile phones for gambling purposes (either at least once last year and at least weekly) between 2013, 2015, and 2019? (3) Across all time points, which factors (gender, age, marital status, children in household, educational level, income, occupational status, country of birth, gambling problem category, number of games gambled, video game participation, and video game problems category) can predict online gambling (either at least once last year and at least weekly)? These questions are important, as answering them can inform gambling operators, regulatory authorities, and treatment agencies about the development of online gambling and identify characteristics of gamblers engaged in online gambling. The potential added valued of the present study to the research field pertains in particular to the use of national representative samples of gamblers, cross-sectional data covering a 6-year period, and the ability to characterize online gamblers on central sociodemographic and gambling characteristics.

## Materials and Methods

### Procedures

The data were collected as part of three national surveys about gaming and gambling problems in Norway. The first survey was conducted during autumn 2013. Here, 24,000 persons aged 16–74 years were randomly selected from the Norwegian Population Registry. They were sent a questionnaire with a prepaid return envelope and an information letter explaining the purpose of the study. Up to two reminders with a new questionnaire were sent to those who did not respond. The respondents could also answer on the Internet. A total of 10,081 answered, of whom 6,034 had gambled during the last year. Another national survey using a similar approach (albeit only based on paper-based questionnaires) was conducted during autumn 2015, entailing a gross sample of 14,000. A total of 5,485 took part in the survey, of whom 3,232 had gambled during the last year. A third survey was conducted in 2019, also using a similar procedure, except that the questions initially could only be answered online. However, both reminders in the 2019 survey included a paper-based questionnaire together with a prepaid return envelope. In the 2019 survey, the gross sample size was 30,000.

A total of 9,248 participated, of whom 5,830 had gambled during the last year. When adjusting for those not able to answer (wrong addresses, dead, abroad, sick, or not able to understand Norwegian), the response rates of the three surveys were 43.6, 40.8, and 32.7%, respectively. In terms of inclusion criteria in the surveys, no other requirement than having an address in Norway and being between 16 and 74 years was enforced. For participating in the present study, the only additional inclusion criterion was that the participant needed to have participated in gambling at least once last year. In order to keep the response rate as high as possible, recommended approaches such as keeping the questionnaire relatively short, printing it in color with a unique ID number, arranging a lottery with gift cards (worth 500 NOK ≈ 50 €) for those who replied, showing researchers' university affiliation, and highlighting confidentiality were emphasized in all three surveys (26). The first two surveys were approved by the Regional Committee for Medical and Health Research Ethics (REK vest 2013/120), whereas the third survey was approved by the Norwegian Center for Research Data (No. 528056).

### Instruments

The same or similar items were used in all three surveys.

#### Sociodemographics

In 2013 and 2015, participants were asked to provide information about *gender* and *age* (in 2019, both were provided by the Norwegian Population Registry). Furthermore, all three surveys included questions about *marital status* (“married/common-law partner” vs. “single/separated/divorced/widow/widower”), *number of cohabitating children* participants had caretaker responsibility for, *highest completed educational level* (“not completed mandatory school,” “mandatory school,” “high school,” “vocational school,” “university/college bachelor's degree,” “university/college master's degree,” or “university/college PhD”), *income before tax* (single item with 11 response alternatives ranging from 0–99,999 NOK to 1,000,000 NOK or more, where each step represented an increase of 100,000 NOK), *occupational status* (“full-time employed,” “part-time employed,” “unemployed,” “student,” “homemaker,” “disability pension/benefit,” “work assessment allowance,” or “retiree”), and *country of birth* (“Norway,” “Nordic country outside Norway,” “Europe outside Nordic country,” “Africa,” “Asia,” “North America,” “South and Central America,” or “Oceania”).

#### Gambling Participation

Participants were asked to report their gambling participation on an item defining gambling (“staking money on the outcome of an event or draw where one can win money”) and asked if they had participated in gambling (in any form) during the last 12 months (“no”/“yes”).

#### Online Gambling

Respondents were asked how often they gambled online using: (a) stationary PC, (b) laptop, (c) tablet, and (d) mobile phone. Each of these four items could be answered: “never,” “less often than once per month,” “about once per month,” “about once per week,” and “about once per day.” Hence, online gambling was in the present paper defined as any type of gambling (e.g., from placing odds online to gamble online interactive games) involving the use of the Internet.

#### Problem Gambling Severity Index

The Problem Gambling Severity Index (PGSI) assesses gambling problems and comprises nine items, each consisting of a description of a problem gambling behavior or a consequence which the participants are asked to rate according to occurrence frequency, ranging from “never” (0) to “always” (3). Based upon the composite score across the nine items, each participant is assigned to one of four gambling categories: Non-problem gambling (sum score of 0), low-risk gambling (sum score of 1 or 2), moderate-risk gambling (sum score of 3–7), and problem gambling (sum score of 8–27) (27). Cronbach's alpha across the nine items was 0.90, 0.88, and 0.91 for the 2013, 2015, and 2019 survey, respectively.

#### Gambling on Specific Types of Games

A list of different types of gambling was provided, and the participants were asked to select the specific types of games they had participated in during the last 12 months. The number and types of games listed changed somewhat across the three surveys due to changes in the gambling marked. In order to compare gambling from survey to survey, only the types of gambling presented in all surveys were included in the present study. These amounted to 17 different games: “paper-based scratch card,” “online-based scratch card,” “bingo in bingo premises,” “data bingo,” “Belago (slot machine in bingo premises),” “online bingo in bingo premises,” “Multix (slot machine),” “gambling on ferries,” “online poker,” “online casino gambling offshore,” “horse racing,” “sport betting, odds games offshore,” “sport betting, odds games state monopolist,” “pool betting,” “number games,” “private gambling,” and “other games.”

#### Participation in Video Gaming

One item defined video gaming (electronic games played on PC/Mac, tablets, mobile phone, or different game consoles like Playstation, Xbox, PS Vita, Nintendo 3DS, and the like), and the respondents were asked if they had participated in video gaming during the last 6 months (“yes”/“no”).

#### Game Addiction Scale for Adolescents

The Game Addiction Scale for Adolescents (GASA) has seven items reflecting the six core addiction (salience, mood modification, tolerance, withdrawal symptoms, conflict, and relapse) components (28) as well as one item related to problems generated by gaming. The response alternatives range from “never” (1) to “very often” (5). According to the instructions, the responses should reflect experiences and behavior during the last 6 months (29). A common approach to identify problem gamers based on GASA is to categorize those scoring 3 or more (i.e., “sometimes” or more often) on 3–6 items as problem video gamers and those scoring 3 or more on all seven items as addicted to video games. In the present study, Cronbach's alpha for the GASA was 0.85, 0.86, and 0.87 for the survey conducted in 2013, 2015, and 2019, respectively.

### Sample

Table 1 presents an overview of the distributions or mean scores and standard deviations for the study variables collected in the three surveys for those who had gambled at least once last year (weighted according to the distribution of age, gender, and county of the general population). Somewhat more men than women were present among the gamblers. Most were married or had a common-law partner, and most lived in households with no children they had caretaker responsibilities for. Bachelor's degree and 400,000–599,999 NOK were the most frequently reported educational and income level, respectively. The majority of the respondents were full-time employed and born in Norway. Among the online gamblers, the largest proportion accessed the Internet *via* a laptop in 2013 (15.4 vs. 12.4% for mobile phone), while the vast majority of online gamblers used a mobile phone (48.7 vs. 16.2% for laptop) for this purpose in 2019. About four in five of the gamblers were non-problem gamblers. Less than half of the gamblers had participated in video gaming during the last 6 months, and more than 90% were categorized as non-gamer/normal gamer.

**Table 1:** Descriptive statistics of study variables in the three (2013, 2015, and 2019) surveys among gamblers.

	**2013**	**2015**	**2019**
**Variable**	**% or mean (SD)**	**% or mean (SD)**	**% or mean (SD)**
*N*	6,034	3,232	5,830
**Gender men/women**	54.0/46.0%	54.6/45.4%	51.5/48.5%
**Age groups**			
16–25 years	11.9%	12.0%	14.7%
26–35 years	18.4%	18.7%	19.7%
36–45 years	21.0%	19.4%	18.6%
46–55 years	19.8%	19.8%	18.9%
56–65 years	17.9%	17.9%	16.1%
66–74 years	11.0%	12.3%	12.0%
**Marital status**			
Married/common-law partner	71.5%	72.0%	68.6%
Single/separated/divorced/widow (er)	28.5%	28.0%	31.4%
**Children in household**			
None	60.7%	62.4%	64.0%
1–2	32.6%	31.2%	29.7%
3 or more	6.7%	6.3%	6.3%
**Highest completed education**			
Not completed mandatory school or mandatory school	8.7%	8.8%	7.5%
High school	24.0%	23.9%	24.1%
Vocational school	23.8%	23.8%	19.3%
Bachelor's degree	29.4%	28.4%	30.9%
Master's degree/PhD	14.1%	15.1%	18.0%
**Income before tax**			
0–199,999 NOK	16.6%	15.9%	17.2%
200,000–399,999 NOK	33.5%	28.3%	22.7%
400,000–599,999 NOK	33.3%	34.5%	33.2%
600,000–799,999 NOK	10.3%	12.8%	15.5%
800,000–999,999 NOK	3.5%	4.5%	6.3%
1,000,000 or more	2.8%	4.0%	5.1%
**Occupational status**			
Full-time employed	59.4%	58.5%	57.9%
Part-time employed	9.3%	10.6%	10.0%
Student	12.0%	7.8%	9.8%
Unemployed/disability pension/work assessment allowance	8.1%	10.7%	10.0%
Homemaker/retiree	11.2%	12.4%	12.3%
**Country of birth**			
Norway	92.1%	92.0%	89.1%
Europe outside Norway/North-America/Oceania	5.5%	5.5%	7.5%
Africa, Asia, South-, and Central-America	2.4%	2.5%	3.4%
**Internet gambling (at least once)**			
Stationary PC	9.1%	9.1%	9.9%
Lap-top	15.4%	14.3%	16.2%
Tablet	6.7%	7.8%	9.8%
Mobile phone	12.4%	17.0%	48.7%
**Gambling category**			
Non-problem gambler	82.1%	81.2%	79.0%
Low-risk gambler	12.9%	13.2%	13.9%
Moderate risk gambler	3.9%	4.0%	4.9%
Problem gambler	1.1%	1.6%	2.1%
**Number of games gambled**	2.4 (1.7)	2.1 (1.7)	2.1 (1.7)
**Gaming participation (yes/no)**	36.2%	36.9%	46.1%
**Gaming category**			
Non-gamer/normal gamer	94.5%	93.8%	90.6%
Problem gamer/game addict	5.5%	6.2%	9.5%

### Statistical Analysis

Data were analyzed with IBM SPSS Statistics, version 25. In all analyses, data were weighed in terms of age, gender, and resident county to adjust for any discrepancies between the full sample and the Norwegian population in the age range of 16–74 years. Adjusted logistic regression analyses (adjusting for gender, age group, and problem gambling category) were conducted in order to investigate whether online gambling of any of the following: stationary PC, laptop, tablet, mobile phone, or any of these platforms, had changed in the period 2013–2015. Year 2015 was used as a reference category. One analysis was performed for having gambled online at least once during the last 12 months (ever), and one analysis was performed for frequent (at least weekly) online gambling. Furthermore, adjusted logistic regression analyses were conducted to investigate characteristics associated with online gambling. Gambled online at least once last year across all modes of Internet access (ever gambled online) and gambled online at least once per week across all modes of Internet access (frequent online gambling) comprised the dependent variables. In both logistic regression models, the independent variables were gender, age group, marital status, children in household, educational level, income, occupational status, country of birth, problem gambling category, number of games gambled, gaming participation, and problem gaming category.

## Results

**Figure 1:**
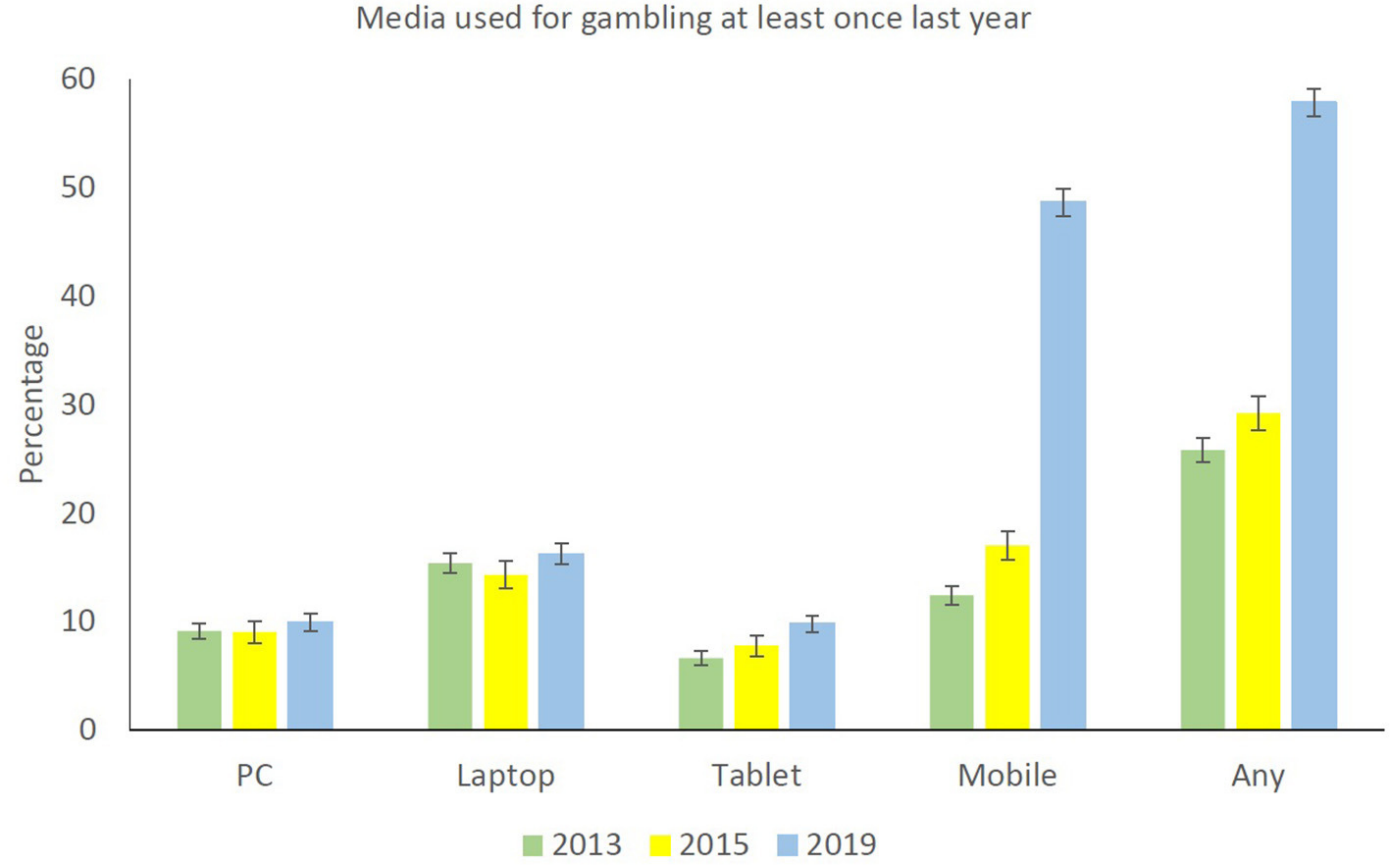
Online gambling at least once last year among gamblers, broken down by year and mode of Internet access for gambling.

**Figure 2:**
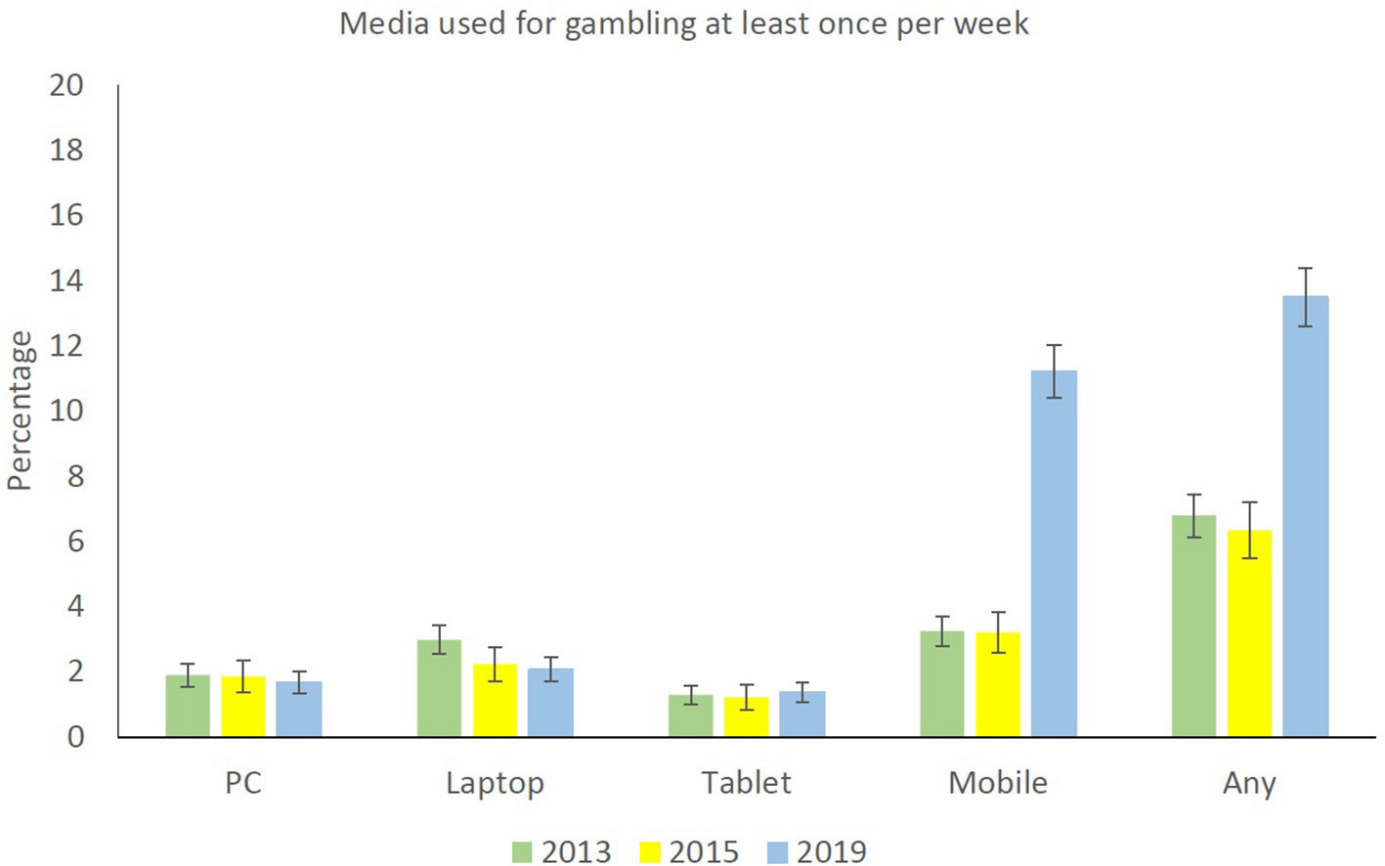
Online gambling at least once per week among gamblers, broken down by year and mode of Internet access for gambling.

The first research question concerned the proportion of gamblers gambling over the Internet (either at least once last year or at least weekly) in 2013, 2015, and 2019. For any mode of access, the probability of gambling online at least once during the last year was lower in 2013 than in 2015 and higher in 2019 than in 2015 (Figure 1 and Table 2). For any mode of access, the probability of gambling online at least weekly was higher in 2019 compared to 2015 (Figure 2 and Table 2).

**Table 2:** Odds ratios for online gambling at least once during the last year and at least weekly by mode of Internet access among gamblers.

		**Online gambling at least once during the least year**		**Online gambling at least weekly**	
**Mode of access**	**Year^**a**^**	**OR^**b**^**	**95% CI**	**OR^**b**^**	**95% CI**
Stationary PC	2013	1.03	0.88–1.21	1.05	0.76–1.45
	2019	1.10	0.94–1.28	0.88	0.63–1.23
Laptop	2013	1.12	0.99–1.28	**1.45**	**1.08–1.94**
	2019	1.13	0.99–1.28	0.87	0.64–1.19
Tablet	2013	0.85	0.72–1.01	1.12	0.75–1.67
	2019	**1.28**	**1.09–1.51**	1.08	0.72–1.60
Mobile phone	2013	**0.67**	**0.59–0.76**	1.03	0.80–1.32
	2019	**5.48**	**4.89–6.14**	**4.00**	**3.21–4.99**
Any	2013	**0.82**	**0.74–0.91**	1.10	0.92–1.32
	2019	**4.01**	**3.62–4.44**	**2.44**	**2.06–2.89**

a *Year 2015 is the reference*.

b *Adjusted for gender, age group, and problem gambling category*.

*Significant findings are shown in bold*.

The second research question concerned the proportion of gamblers gambling over the Internet broken down by mode of access. For gambling at least once last year, no changes by year were found for either stationary PC or laptop. The probability of gambling online on a tablet was, however, significantly higher in 2019 than in 2015. For mobile phone, the probability of frequently gambling online was significantly higher in 2019 than 2015 (Figure 1 and Table 2). For online gambling at least weekly the probability of gambling on a laptop was lower in 2015 than in 2013, wheres the probability of at least weekly online gambling using a mobile phone was higher in 2019 than in 2015 (Figure 2 and Table 2). The third and last research question addressed differences between online and non-online gamblers. Table 3 presents the finding for the results of the logistic regression analysis predicting online gambling at least once during the last year. The model was significant (χ^2^ = 2669.6, *df* = 30, *p* < 0.001), and the predictors explained between 17.9% (Cox and Snell *R*^2^) and 24.2% (Nagelkerke *R*^2^) of the variance. The model with the intercept only correctly classified 60.1% of the respondents, whereas the model including all predictors correctly classified 70.6% of the respondents. Significant predictors of online gambling at least once last year were male gender and young age. Those with three or more children in the household had a lower probability of online gambling at least once during the last year than those with no children in the household. Those with high school or bachelor's degree had a higher probability of online gambling at least once during the last year than those not having completed mandatory school or with mandatory school only. Those with higher income than the lowest class (0–199,999 NOK) had a higher probability of online gambling at least once during the last year. Compared to respondents with a full-time position, those working part-time, being unemployed/on disability pension/on work assessment allowance, and homemakers/retirees had a higher probability of online gambling at least once during the last year. Country of birth was unrelated to online gambling at least once during the last year. Those categorized as a low-risk gambler, moderate-risk gambler, and problem gambler all had a higher probability of online gambling at least once during the last year compared to those in the non-problem gambler category. Number of games gambled was positively associated with online gambling at least once during the last year. Participating in video gaming (as opposed to not participating) during the last 6 months was associated with an increased probability of online gambling at least once during the last year, whereas the category of video game problems was unrelated to online gambling at least once during the last year.

**Table 3:** Results of logistic regression analysis predicting gambled online at least once last year and gambled online at least once per week among gamblers.

	**Gambled online at least once last year**		**Gambled online at least once per week**	
**Independent variable**	**OR**	**95% CI**	**OR**	**95% CI**
**Gender**				
Woman^a^	1.00		1.00	
Man	**1.62**	**1.48–1.76**	**1.97**	**1.70–2.29**
**Age**				
16–25 years	**2.92**	**2.31–3.69**	0.74	0.50–1.09
26–35 years	**2.64**	**2.15–3.24**	1.09	0.79–1.51
36–45 years	**2.09**	**1.70–2.58**	1.37	0.99–1.90
46–55 years	**1.76**	**1.44–2.14**	**1.57**	**1.15–2.14**
56–65 years	**1.31**	**1.09–1.58**	1.22	0.91–1.64
66–74 years^a^	1.00		1.00	
**Marital status**				
Married/common-law partner^a^	1.00		1.00	
Single/separated/divorced/widow (er)	1.03	0.93–1.13	1.08	0.93–1.26
**Children in household**				
None^a^	1.00		1.00	
1–2 children	0.95	0.86–1.05	0.97	0.83–1.14
3 or more children	**0.77**	**0.65–0.92**	0.87	0.66–1.15
**Education**				
Not completed mandatory/mandatory school^a^	1.00		1.00	
High school	**1.36**	**1.15–1.60**	1.13	0.89–1.44
Vocational school	1.10	0.93–1.31	0.84	0.66–1.08
Bachelor's degree	**1.37**	**1.16–1.62**	0.90	0.71–1.16
Master's degree/PhD	0.92	0.77–1.11	**0.67**	**0.50–0.90**
**Income**				
0–199,999 NOK^a^	1.00		1.00	
200,000–399,999 NOK	**1.44**	**1.23–1.69**	**1.48**	**1.15–1.92**
400,000–599,999 NOK	**2.18**	**1.82–2.60**	**2.00**	**1.50–2.67**
600,000–799,999 NOK	**3.07**	**2.50–3.77**	**2.11**	**1.52–2.92**
800,000–999,999 NOK	**3.28**	**2.57–4.19**	**2.68**	**1.84–3.90**
1,000,000 or more	**3.29**	**2.54–4.27**	1.49	0.96–2.30
**Occupational status**				
Full-time employed^a^	1.00		1.00	
Part-time employed	**1.16**	**1.00–1.35**	1.02	0.79–1.32
Student	1.01	0.84–1.21	0.91	0.66–1.24
Unemployed/disability pension/work assessment allowance	**1.51**	**1.29–1.77**	**1.42**	**1.14–1.86**
Homemaker/retiree	**1.21**	**1.02–1.44**	**1.37**	**1.06–1.78**
**Country of birth**				
Norway^a^	1.00		1.00	
Europe outside Norway/North America/Oceania	0.93	0.79–1.10	0.80	0.61–1.04
Africa, Asia, South America, and Central America	1.08	0.85–1.39	1.25	0.90–1.74
**Gambling category**				
Non-problem gambler^a^	1.00		1.00	
Low-risk gambler	**2.15**	**1.92–2.41**	**2.36**	**2.02–2.76**
Moderate-risk gambler	**3.47**	**2.79–4.31**	**3.74**	**2.99–4.70**
Problem gambler	**3.04**	**2.09–4.41**	**5.37**	**3.81–7.71**
**Number of games gambled**	**1.34**	**1.31–1.38**	**1.22**	**1.18–1.26**
**Played video games last 6 months**				
No^a^	1.00		1.00	
Yes	**1.52**	**1.41–1.69**	1.08	0.93–1.25
**Gaming problems**				
Non-gamer/normal gamer^a^	1.00		1.00	
Problem gamer/game addict	1.04	0.88–1.22	0.98	0.78–1.23

a *Comprise the reference category*.

*Significant findings are shown in bold*.

Table 3 also presents the findings for the results of the logistic regression analysis predicting frequent (at least once per week) online gambling. The model was significant (χ^2^ = 1039.2, *df* = 30, *p* < 0.001), and the predictors explained between 7.4% (Cox and Snell *R*^2^) and 15.8% (Nagelkerke *R*^2^) of the variance. The model with the intercept only correctly classified 90.5% of the respondents. Classification was not improved by the model including all predictors. Men gambled more frequently online than women. The respondents in the age range of 46–55 years had a higher probability of frequent online gambling compared to ones in the age range of 66–74 years. Marital status and children in the household were unrelated to frequent online gambling. Those with a master's degree/PhD had a lower probability of frequent online gambling than those who had not completed or only completed mandatory school. People earning 200,000–999,999 NOK had a higher probability of frequent online gambling than those with the lowest (0–199,999 NOK) income. Those being unemployed/on disability pension/on work assessment allowance as well as homemakers/retirees had a higher probability of frequent online gambling compared to the reference group (full-time employed). Country of birth was not related to frequent online gambling. Low-risk gamblers, moderate-risk gamblers, and problem gamblers all had a higher probability of frequent online gambling compared to non-problem gamblers. Number of games gambled increased the probability of frequent online gambling. Neither involvement with video games nor gaming problems were associated with frequent online gambling.

Taken together, online gambling, especially on mobile phones, has increased significantly from 2013 to 2019. Consistent predictors of online gambling (both ever and frequent) were male gender, young age, earning high income, not working or studying, having gambling problems, and number of games gambled.

In the 2013 survey, 6.3% responded *via* Internet and 93.7% responded *via* a paper questionnaire. Of these, 50.4 and 25.2% (χ^2^ = 111.9, *df* = 1, *p* < 0.001, continuity correction) had gambled online, respectively. The data collection of the 2015 survey was exclusively conducted *via* paper-based questionnaires. In the 2019 survey, 65.6% responded *via* Internet and 34.4% responded *via* a paper questionnaire. Of these, 62.9 and 48.1% (χ^2^ = 118.1, *df* = 1, *p* < 0.001, continuity correction) had gambled online, respectively.

## Discussion

Overall, online gambling among gamblers had increased during the last 6 years in Norway, both in terms of ever (at least once during the last 12 months) and frequent (at least once per week) online gambling. This increase is attributable to increased online gambling on mobile phones, which now, by far, seems to be the most used mode of Internet access by gamblers. Another study showed however that online gambling *via* computers was the most frequent online gambling mode (20), whereas a more recent study of help-seeking gamblers attested to mobile phones as the preferred mode of Internet access for gambling purposes (7). Taking publication year into consideration, these findings overall suggest that mobile phone seems to have become the prevailing mode of accessing the Internet for gambling purposes. This development may be worrisome as, in line with the accessibility hypothesis, those gambling online on mobile phones report more often gambling problems than those who gamble on a computer (30).

Online gambling (ever and frequent) was more common among men than women. This is in line with several other studies (9, 13, 19, 20, 31) and most likely reflects that men generally are more involved in gambling than women (32). Young subjects had a higher probability of gambling online compared to older ones (especially at least once during the last 12 months). This also run tandem with previous findings (9, 19, 20, 31) and suggests that younger people in general are more familiar with Internet use than older people (33) and may also be more attracted to the games available there. For frequent online gambling, the only significant finding related to age was that the age group 46–55 years had a higher probability of such gambling than those 66–74 years old. Unlike other studies, marital status was unrelated to online gambling. One explanation to this is that the present study controlled for several sociodemographic variables simultaneously. Those with three or more children in the household had a lower probability of ever gambled online during the last year compared to those with no children in the household. This may imply that high childcare responsibility load, probably due to time constraints, prevents online gambling. Those with a high school education and a bachelor's degree had a higher probability of online gambling (at least once during the last year) than those not having completed any education beyond mandatory school. Similarly, those with a master's degree exhibited a lower probability for engaging in frequent online gambling. These findings are in accordance with a study from Sweden showing that a higher proportion of those with medium (as opposed to low and high) level of education gambled online (31). One explanation to this finding is that those with low education are more Internet-illiterate than those with a higher education (34). Those with the highest education were less inclined to frequent online gambling compared to those not having completed any education beyond mandatory school. This may reflect that the former group is less interested in online gambling due to being less influenced by cognitive biases (35) and may thus perceive gambling in a more realistic way. Those in the lowest income class had a lower probability of Internet gambling (both ever and frequent) than those with higher incomes. This runs counter with two other studies showing no relationship between income and online gambling (9, 36). The present finding most likely reflects that people with a low income have limited amounts of money to spend gambling. Regarding occupational status, the results showed that unemployed, people on disability pension, work assessment allowance, homemakers, and retirees were overrepresented among online gamblers (both ever and frequent) compared to full-time employees. The reason for this is not clear, but it may reflect that those in the former groups have more free or available time to gamble than those employed full-time (19). Country of birth was unrelated to online gambling. Overall, the most consistent predictor of online gambling was gambling category, showing that both low-risk gamblers, moderate-risk gamblers, and problem gamblers had a higher probability of online gambling (both ever and frequent) than non-problem gamblers (while controlling for all other variables including number of games gambled). This is in contrast to a former study showing that gambling problems were inversely related to online gambling when controlling for the number of games gambled (15). The discrepancy between the current finding and the findings of Philander and MacKay (15) may relate to the year of the surveys, as the data of Philander and MacKay's (15) were collected in 2010, while the current study's data were collected in 2013, 2015, and 2019. It is conceivable that online gambling was more uncommon and less advanced in 2010 and that the association between problem gambling and online gambling in 2010 could be explained by problem gamblers seeking out a larger number of different games (online as well as offline). By 2013 and later, however, online gambling has become more common including more advance games containing “addictive features.” Thus, the association between problem gambling and online gambling can no longer be explained solely by the number of games played and may instead perhaps be explained by features of online gambling facilitating the development of problem gambling. The finding showing that those with gambling problems were more involved (both ever and frequent) with online gambling than non-problem gamblers is further in line with the majority of studies on this topic (8–14). Having played video games during the last 6 months was associated with an increased probability of having gambled online at least once during the last year but was unrelated to frequent online gambling. This may suggest that a common denominator between gaming and online gambling is the use of relevant technology. The fact that gaming problems were not related to the probability of online gambling, neither ever nor frequent, supports this notion and does not support previous findings showing a positive relationship between online gambling and Internet gaming disorder (23).

It may appear contradictory that both high income and unemployment of some sort (e.g., disability pension) were associated with online gambling. However, each association was adjusted for all of the other included variables, thus it makes sense that individuals who are not at work may engage in more online gambling (and gambling in general) when income level is held constant and *vice versa*—that higher income may be associated with more gambling when employment status is held constant. Both in the 2013 and in the 2019 survey, a correspondence between answering format (*via* paper or Web) and participation in online gambling (no vs. yes) was found. This seems reasonable and suggests that people's general online usage is associated with online gambling. Still, as the sample was drawn from the National Population Registry, the mode of answering should not influence the overall representativeness of the sample as a whole.

### Limitations and Strengths

A limitation of the present study is the mediocre response rates, which may limit the generalizability of the findings, although it could be argued that the response rates are reasonable, taking the general falling response rate to surveys worldwide into account (37). The cross-sectional design of the study, although based on three surveys conducted over a 6-year span, prevents conclusions about directionality and causality. Regarding the numbers of games controlled for, it should be noted that some categories were broad and contained more than one game (e.g., number games), whereas other games were represented by more than one category (sports betting offshore or with state monopolist). Another limitation is that the present study did not differentiate between online gambling in terms of just placing bets (e.g., sports betting and number games) and online gambling (e.g., online casino games) where the games themselves unfold on the Internet. Still, in both cases, it is arguable that online gambling increases availability, hence the current operationalization is justifiable from such point of view. The second regression model explained less variance than the first. This most likely reflects differences in base rate (in this case, proportion of those who have gambled online) between the two models (0.391 and 0.093), as the outcome in cases where the base rate is close to 0 or 1 is already much determined in contrast to outcomes in which the base rate is close to 0.5 (38). Strengths of the present study are the high number of respondents, the representative samples of gamblers drawn from the National Population Registry, and the use of validated instruments to assess gambling (27), as well as gaming problems (29). The fact that the relationship with online gambling and relevant correlates was analyzed using a multivariable approach, controlling for several confounders is also an asset of the present study. As far as we know, the present study is the first elucidating change over time in terms of online gambling in representative samples.

### Conclusions

Among gamblers, online gambling, especially on mobile phones, has increased significantly from 2013 to 2019. Since the consistent predictors of online gambling (both ever and frequent) were found to be male gender, young age, earning high income, not working or studying, having gambling problems, and number of games gambled, responsible gambling initiatives aimed at preventing or minimizing harm related to online gambling (e.g., responsible gambling tools) should thus target those in these groups. In terms of policy implications, the results showing a significant increase in online gambling suggest that gambling operators should use this as an opportunity to increase their focus on mandatory registered gambling and responsible gambling initiatives, as both are more feasible to implement in online compared to offline gambling settings (39).

## Data Availability Statement

The raw data supporting the conclusions of this article will be made available by the authors, without undue reservation.

## Ethics Statement

The studies involving human participants were reviewed and approved by Regional Committee for Medical and Health Research Ethics (REK vest 2013/120). Written informed consent for participation was not required for this study in accordance with the national legislation and the institutional requirements.

## Author Contributions

SP, RM, AM, and EE designed the study and collected the data. SP drafted the first version of the manuscript and conducted the analyses. RM, AM, JE, PK, and EE critically revised the manuscript. All authors approved the final version of the manuscript submitted for publication.

## Conflict of Interest

The authors declare that the research was conducted in the absence of any commercial or financial relationships that could be construed as a potential conflict of interest.
